# Microstructure and Mechanical Properties of the Powder Metallurgy Nb-16Si-24Ti-2Al-2Cr Alloy

**DOI:** 10.3390/ma17164155

**Published:** 2024-08-22

**Authors:** Feng Wen, Wentao Liu, Ao Fu, Qianli Huang, Jian Wang, Yuankui Cao, Jingwen Qiu, Bin Liu

**Affiliations:** 1 China North Nuclear Fuel Co., Ltd., Baotou 014035, China; 2 State Key Laboratory of Powder Metallurgy, Central South University, Changsha 410083, Chinacaoyuankui@csu.edu.cn (Y.C.); 3 School of Materials Science and Engineering, Hunan University of Science and Technology, Xiangtan 411201, China; jingwen.qiu@hnust.edu.cn

**Keywords:** Nb-Si alloy, hot pressing, microstructure, mechanical property, fracture toughness

## Abstract

The Nb-16Si-24Ti-2Al-2Cr alloy was prepared by plasma rotating electrode process (PREP) technology and the hot-pressing (HP) method, and the effects of sintering temperature on the microstructure, mechanical properties and fracture behavior were investigated. The HP alloys sintered at temperatures below 1400 °C are composed of Nbss (Nb solid solution), Nb_3_Si and Nb_5_Si_3_ phases. When the sintering temperature reaches 1450 °C, the Nb_3_Si phase is completely decomposed into Nbss and Nb_5_Si_3_ phases. Meanwhile, the microstructure coarsens significantly. Compared with the cast alloy, the HP alloy shows better mechanical properties. The fracture toughness of the alloy sintered at 1400 °C reaches 20.2 MPa·m^1/2^, which exceeds the application threshold. The main reason for the highest fracture toughness is attributed to the decomposition of large-sized brittle Nb_3_Si phase and the formation of a fine microstructure, which greatly increases the number of phase interfaces and improves the chance of crack deflection. In addition, the reduction in the size and content of silicides also reduces their plastic constraints on the ductile Nbss phase.

## 1. Introduction

With the rapid development of the aviation industry in recent years, there is a huge demand for high-thrust turbine engines for advanced heat-resistant materials that can withstand higher temperatures. As an intermetallic compound, Nb_5_Si_3_ has excellent comprehensive properties such as a high melting point, high strength and low density, showing great potential in the thermal components of advanced propulsion systems [1,2,3,4]. However, Nb_5_Si_3_ exhibits poor machinability (e.g., cutting, turning and drilling) and low fracture toughness at room temperature, which seriously limits its practical application [5,6]. To solve the above problems, the Nbss/Nb_5_Si_3_ alloy was designed based on the Nb-Si binary phase diagram [7,8,9], trying to combine the ductility of Nbss (Nb solid solution) and the strength of Nb_5_Si_3_. The Nbss/Nb_5_Si_3_ alloy has been shown to be thermochemically and morphologically stable at 1500 °C for at least 100 h [10]. Therefore, the Nbss/Nb_5_Si_3_ alloy is expected to become an ultra-high-temperature structural material.

Vacuum arc melting and directional solidification are commonly used methods to fabricate the Nbss/Nb_5_Si_3_ alloy [11,12,13,14,15]. However, due to the extremely high melting point and brittleness, the Nbss/Nb_5_Si_3_ alloy prepared by the above methods has problems such as component segregation and uncontrollable structure [16,17,18]. In addition, the as-cast Nbss/Nb_5_Si_3_ alloy has a coarse structure, with a grain size of tens of microns, and fails by cleavage, thus affecting the mechanical properties [3,19,20,21]. Although the addition of alloying elements improves the fracture toughness to a certain extent, there is still a large gap from the application threshold of 20 MPa·m^1/2^. Zhao et al. [22] prepared a series of Nb-16Si-22Ti-xMn alloys by arc melting and found that although the addition of Mn can improve the fracture toughness, the fracture toughness of the optimal component is only 8.07 MPa·m^1/2^. Powder metallurgy technology is considered to be an effective method for preparing high-performance alloys [23,24,25,26,27], which can optimize the morphology, size and distribution of the Nbss and Nb_5_Si_3_ phases, thereby achieving a balance between strength and toughness in the Nbss/Nb_5_Si_3_ alloy. Liu et al. [28] found that the fracture toughness of the Nb-16Si alloy prepared by spark plasma sintering reached 12.4 MPa·m^1/2^, which was significantly better than that of the as-cast alloy with the same composition. Meanwhile, the powder metallurgy method can refine the grain size. As the grain size decreases, the fracture mode of the Nb-16Si alloy changes from cleavage fracture to a mixed mode of dimple, tear and cleavage. Mendiratta et al. [29] also found that the fracture toughness of the Nb-16Si alloy prepared by hot extrusion increased from 5.4 MPa·m^1/2^ in the cast state to 12.6 MPa·m^1/2^.

Herein, a novel Nbss/Nb_5_Si_3_ alloy (Nb-16Si-24Ti-2Al-2Cr, at.%) was synthesized by plasma rotating electrode process (PREP) technology and the hot-pressing (HP) method. The minor addition of Al, Cr and Ti in the Nb-16Si-24Ti-2Al-2Cr alloy can improve its fracture toughness and oxidation resistance [18,30]. The effect of sintering temperature on the microstructure, mechanical properties and fracture behavior of the HP Nb-16Si-24Ti-2Al-2Cr alloy were systematically analyzed.

## 2. Experimental Procedure

**Figure 1:**
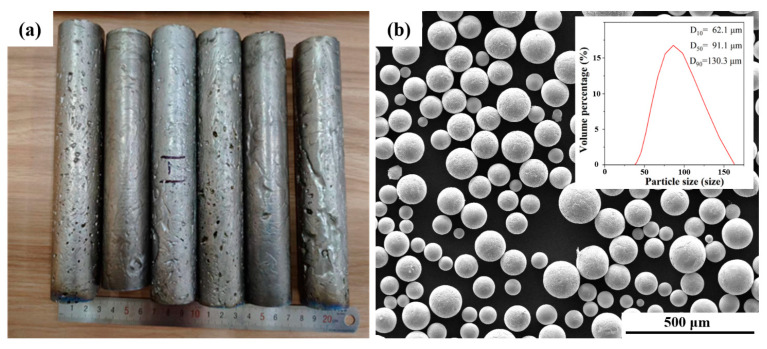
(**a**) CCLM Nb-16Si-24Ti-2Al-2Cr ingots and (**b**) PREP Nb-16Si-24Ti-2Al-2Cr powders.

Nb-16Si-24Ti-2Al-2Cr ingots were prepared by the cold-crucible levitation melting (CCLM) method and were remelted three times to ensure the uniform composition. The ingots were machined into electrode rods of φ 33 mm × 200 mm (Figure 1a) and then prepared into the Nb-16Si-24Ti-2Al-2Cr spherical powders by the PREP method. The sieved powders with a particle size range from 45 μm to 150 μm were used as the raw material for hot-pressing sintering. Figure 1b shows the powder morphology and particle size distribution. The powders have high sphericity, and the average particle size is ~91.1 μm. The hot-pressing sintering experiment was carried out on FHP-828 equipment at different sintering temperature (1200 °C, 1250 °C, 1300 °C, 1350 °C, 1400 °C and 1450 °C) under Ar atmosphere. The sintering pressure, sintering time and heating rate were 50 MPa, 10 min and 100 °C/min, respectively. For convenience, the samples sintered at 1200 °C, 1250 °C, 1300 °C, 1350 °C, 1400 °C and 1450 °C are named as HP-1200, HP-1250, HP-1300, HP-1350, HP-1400 and HP-1450, respectively.

Phase composition was analyzed by a Siemens-D5000 X-ray diffraction (XRD) (Munich, Germany). Microstructure and fracture morphology were observed by a Quanta-250FEG scanning electron microscope (SEM) (Houston, TX, USA). Vickers hardness was measured by a BUEHLER-5104 microhardness tester (Chipping Norton, NSW, Australia) under a load of 500 g for 15 s. Each test was repeated at least seven times. A compression experiment was carried out on an Instron-8802 universal testing machine (Norwood, MA, USA) at a strain rate of 0.005 s^−1^. Fracture toughness was determined by an Instron-3369 universal testing machine by the three-point method at a strain rate of 0.2 mm/min. Cylindrical specimens (Φ 6 mm × 9 mm) and strip specimens (20 mm × 4 mm × 2 mm) were used for compression and bending tests, respectively. Each test was repeated at least three times.

## 3. Results and Discussion

**Figure 2:**
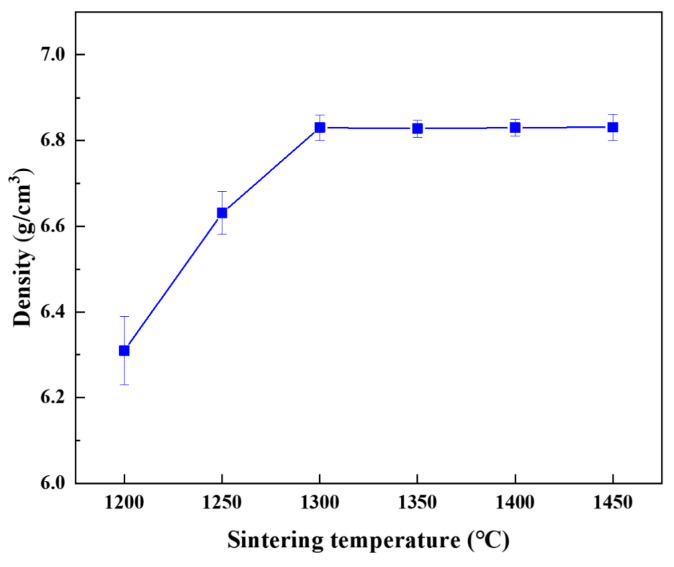
Densities of the HP Nb-16Si-24Ti-2Al-2Cr alloy at different sintering temperatures.

Figure 2 shows the variation curve of density with sintering temperature for the Nb-16Si-24Ti-2Al-2Cr alloy. It can be found that the density of the HP-1200 alloy is only 6.307 g/cm^3^. The sintering temperature of 1200 °C is not adequate to achieve a fully dense microstructure. As the sintering temperature increases, the density of the Nb-16Si-24Ti-2Al-2Cr alloy increases rapidly. A high sintering temperature can promote the fusion of powders and the closure of micropores. When the sintering temperature is 1300 °C, the density reaches 6.831 g/cm^3^, indicating that the alloy has basically achieved densification.

**Figure 3:**
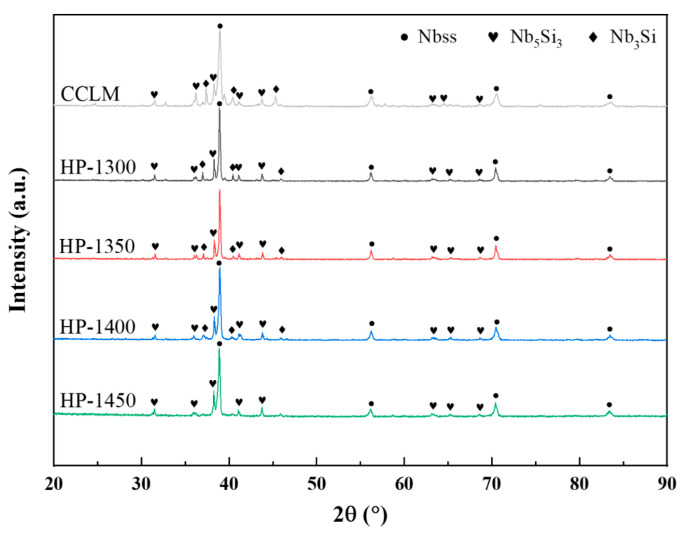
XRD patterns of the CCLM Nb-16Si-24Ti-2Al-2Cr alloy and HP Nb-16Si-24Ti-2Al-2Cr alloy at different sintering temperatures.

Figure 3 shows the XRD patterns of the CCLM Nb-16Si-24Ti-2Al-2Cr alloy and HP Nb-16Si-24Ti-2Al-2Cr alloy. Obviously, the CCLM alloy is mainly composed of three phases: Nbss (JCPDS, 35-0789), Nb_3_Si (JCPDS, 22-0763) and Nb_5_Si_3_ (JCPDS, 24-813). Similar to the CCLM alloy, the HP-1300 alloy consists of Nbss, Nb_3_Si and Nb_5_Si_3_ phases. As the sintering temperature rises to 1350 °C and 1400 °C, the phase composition of the HP-1350 and HP-1400 alloys remains unchanged, still consisting of three phases: Nbss, Nb_3_Si and Nb_5_Si_3_. However, when the sintering temperature further increases to 1450 °C, the diffraction peak of the Nb_3_Si phase disappears, indicating that the higher sintering temperature promotes the complete decomposition of the metastable Nb_3_Si phase, and the HP-1450 alloy consists of two phases: Nbss and Nb_5_Si_3_.

**Figure 4:**
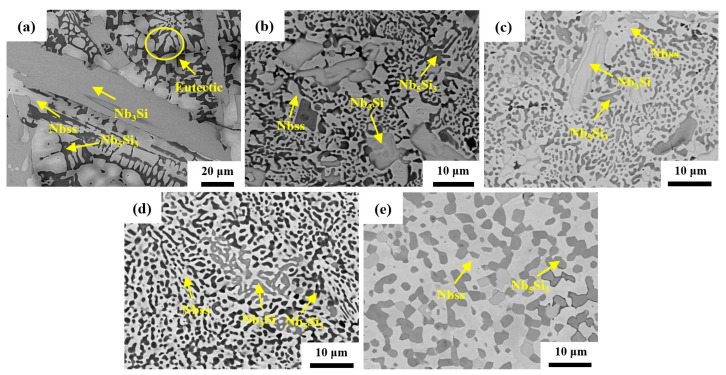
BSE images of (**a**) the CCLM Nb-16Si-24Ti-2Al-2Cr alloy and HP Nb-16Si-24Ti-2Al-2Cr alloy sintered at (**b**) 1300 °C, (**c**) 1350 °C, (**d**) 1400 °C and (**e**) 1450 °C.

Figure 4 shows the SEM images of the CCLM Nb-16Si-24Ti-2Al-2Cr alloy and HP Nb-16Si-24Ti-2Al-2Cr alloy. It can be found that the CCLM alloy consists of a light Nbss phase, a gray Nb_3_Si phase and a black Nb_5_Si_3_ phase (Figure 4a). Compared with the CCLM alloy, the microstructure of the HP-1300 alloy is refined, and a large number of fine Nb_5_Si_3_ particles are uniformly distributed in the Nbss matrix. Meanwhile, the volume fraction of the Nb_5_Si_3_ phase decreases significantly (Figure 4b). As the sintering temperature increases, the size and volume fraction of the Nb_3_Si phase gradually decrease (Figure 4c). The HP-1400 alloy exhibits a typical eutectic structure, with fine Nb_3_Si and Nb_5_Si_3_ particles uniformly distributed in the Nbss matrix (Figure 4d). As the sintering temperature increases to 1450 °C, the Nb_3_Si phase disappears completely, and the Nb_5_Si_3_ phase coarsens (Figure 4e).

**Figure 5:**
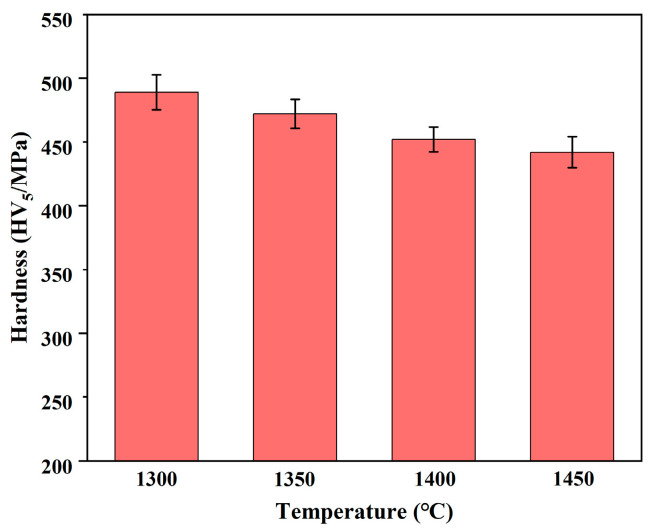
Hardness of the Nb-16Si-24Ti-2Al-2Cr alloy at different sintering temperatures.

Figure 5 shows the microhardness of the HP Nb-16Si-24Ti-2Al-2Cr alloy. The microhardness of the HP-1300, HP-1350, HP-1400 and HP-1450 alloys is 496 HV, 478 HV, 461 HV and 452 HV, respectively. The decrease in microhardness is related to the size and volume fraction of silicides in the Nb-16Si-24Ti-2Al-2Cr alloy. As the sintering temperature increases, the volume fraction of the silicides decreases, and thus the microhardness of the Nb-16Si-24Ti-2Al-2Cr alloy decreases continuously. When the sintering temperature reaches 1450 °C, the volume fraction of the silicides is the lowest, and the microstructure coarsens significantly, resulting in the lowest microhardness of the HP-1400 alloy.

**Figure 6:**
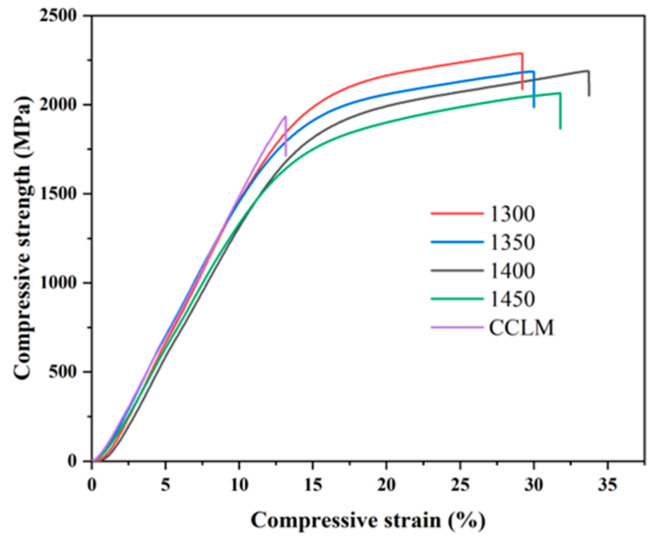
Compression strain–stress curves of the CCLM Nb-16Si-24Ti-2Al-2Cr alloy and HP Nb-16Si-24Ti-2Al-2Cr alloy at different sintering temperatures.

Figure 6 shows the compression curves of the CCLM Nb-16Si-24Ti-2Al-2Cr alloy and HP Nb-16Si-24Ti-2Al-2Cr alloy. It can be found that the CCLM alloy fractured before yielding. Compared with the CCLM alloy, the HP-1300 alloy exhibits better compression performance, with a yield strength, compressive strength and fracture strain of 1644.6 MPa, 2286.7 MPa and 29.2%, respectively. As the sintering temperature increases, the strength of the Nb-16Si-24Ti-2Al-2Cr alloy decreases slightly, but the plasticity increases significantly. The decrease in strength and increase in ductility are mainly attributed to the decrease in the brittle silicides and the increase in the ductile Nbss phase caused by the increase in the sintering temperature [31].

**Figure 7:**
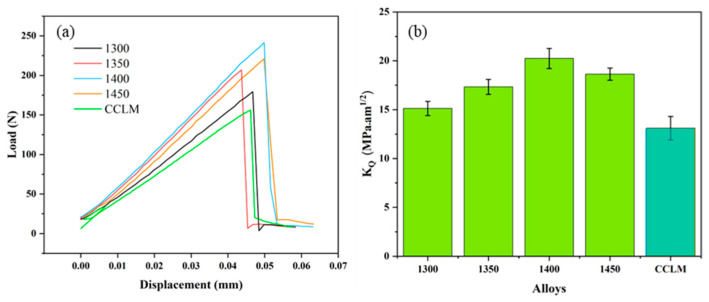
(**a**) Typical load–displacement curves and (**b**) fracture toughness of the CCLM Nb-16Si-24Ti-2Al-2Cr alloy and HP Nb-16Si-24Ti-2Al-2Cr alloy at different sintering temperatures.

Figure 7 shows the load–displacement curve of the fracture toughness for the CCLM Nb-16Si-24Ti-2Al-2Cr alloy and HP Nb-16Si-24Ti-2Al-2Cr alloy. All curves show a trend of first rising and then falling. When the curve reaches the maximum load, the crack expands rapidly and eventually causes the material to fracture. The maximum load of the CCLM alloy is only 156.2 N, and the corresponding fracture toughness is 13.1 MPa·m^1/2^. The abundant presence of the brittle Nb_3_Si phase and coarse microstructure in the CCLM alloy are detrimental to the fracture toughness [32,33]. For the HP alloy, the maximum load increases from 179.3 N to 241.5 N and the fracture toughness increases from 15.1 MPa·m^1/2^ to 20.2 MPa·m^1/2^ as the sintering temperature increases from 1300 °C to 1400 °C. This is due to the fact that the brittle Nb_3_Si phase decreases and the ductile Nbss phase increases as the sintering temperature increases. When the sintering temperature is 1450 °C, the maximum load and fracture toughness are 220.7 N and 18.6 MPa·m^1/2^, respectively. Although the volume fraction of the Nbss phase for the HP-1450 alloy reaches the maximum, the microstructure coarsens significantly. Therefore, the fracture toughness of the HP-1450 alloy is the highest, which is 54% higher than that of the CCLM alloy.

**Figure 8:**
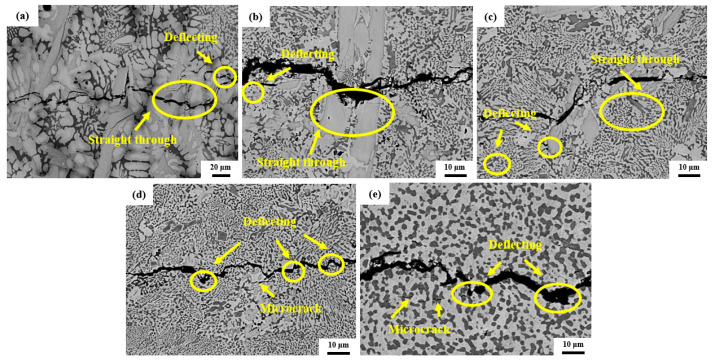
Crack paths of (**a**) the CCLM Nb-16Si-24Ti-2Al-2Cr alloy and HP Nb-16Si-24Ti-2Al-2Cr alloy sintered at (**b**) 1300 °C, (**c**) 1350 °C, (**d**) 1400 °C and (**e**) 1450 °C.

Figure 8 shows the SEM images of the CCLM Nb-16Si-24Ti-2Al-2Cr alloy and HP Nb-16Si-24Ti-2Al-2Cr alloy after fracture. For the HP alloy, the crack propagates in a zigzag path through the Nbss phase to provide toughness. When the crack encounters the Nb_5_Si_3_ phase during the expansion process, it mainly expands in the following two ways. One is that the crack bypasses the Nbss phase and expands along the Nb_5_Si_3_/Nbss phase interface, and this expansion mechanism can enhance the fracture toughness. The other is that the crack passes directly through the Nb_5_Si_3_ phase, in which case the contribution of the Nb_5_Si_3_ phase to the fracture toughness is relatively small. In addition, there are a large number of microcracks in the silicides around the main crack. The formation of microcracks can absorb the energy during the crack propagation and change the stress state at the crack tip, thus inhibiting the propagation of the main crack [34,35]. The bending of cracks and the formation of microcracks can consume more strain energy at the crack tip and are the main toughening mechanisms of the Nb-Si alloys [36,37]. For the HP alloy sintered at temperatures below 1400 °C, the cracks directly pass through the coarse Nb_3_Si particles, which is detrimental to fracture toughness. When the sintering temperature rises to 1400 °C, the decomposition of the Nb_3_Si phase leads to an increase in the volume fraction of the Nbss phase, and the cracks can better propagate in the Nbss matrix, leading to an improvement in fracture toughness. As the sintering temperature is further increased to 1450 °C, the excessively high volume fraction of the Nb_5_Si_3_ phase leads to an increase in the crack paths passing through the Nb_5_Si_3_ particles, which is not conducive to the improvement of fracture toughness.

**Figure 9:**
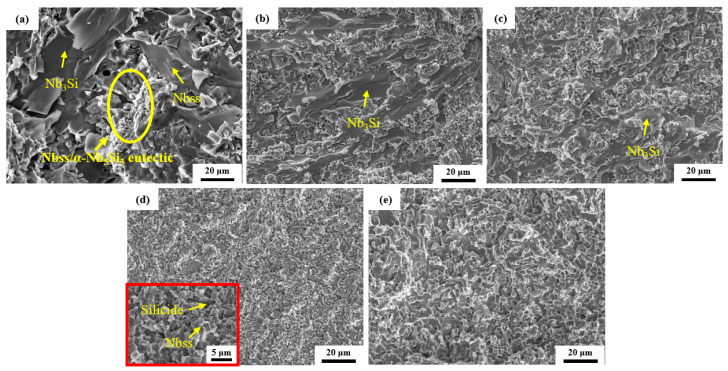
Fracture morphology of (**a**) the CCLM Nb-16Si-24Ti-2Al-2Cr alloy and HP Nb-16Si-24Ti-2Al-2Cr alloy sintered at (**b**) 1300 °C, (**c**) 1350 °C, (**d**) 1400 °C and (**e**) 1450 °C.

Figure 9 shows the fracture morphologies of the CCLM Nb-16Si-24Ti-2Al-2Cr alloy and HP Nb-16Si-24Ti-2Al-2Cr alloy. It can be seen from the fracture morphology of the CCLM alloy that the fracture surface of the silicides is very smooth, showing typical brittle fracture characteristics. The fracture surface of the Nbss phase presents two morphologies. The fracture mode of the Nbss phase is closely related to the size. The smaller Nbss phase is dominated by tearing mode, while the larger Nbss phase is characterized by cleavage fracture with a river pattern [38,39]. Compared with the CCLM alloy, the brittle fracture characteristics of the HP-1300 alloy surface are significantly reduced, which means that the fracture toughness is improved. As the sintering temperature increases, the tearing area increases significantly. The tear marks are distributed all over the surface of the HP-1400 alloy, which also proves its highest fracture toughness.

**Figure 10:**
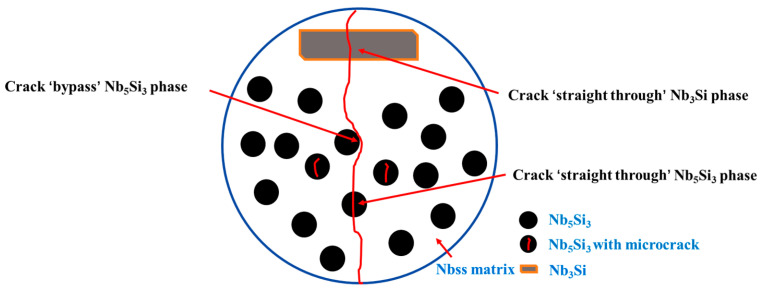
Schematic diagram of crack propagation of the HP Nb-16Si-24Ti-2Al-2Cr alloy.

In order to better understand the toughening mechanism of the HP Nb-16Si-24Ti-2Al-2Cr alloy, Figure 10 illustrates the schematic diagram of crack propagation during deformation process. It can be found that the crack directly passes through the Nb_3_Si phase without deflection due to the high brittleness of the Nb_3_Si phase. There is a probability that the crack will deflect when passing through the interface between Nb_5_Si_3_ and Nbss phases. Therefore, the increase in the phase interface can increase the chance of crack deflection and thus absorb more energy [40,41]. The ductility Nbss phase can coordinate deformation and relieve stress concentration during fracture, and high volume fraction of the Nbss phase is beneficial to the improvement of fracture toughness [42]. As the sintering temperature increases to 1400 °C, the size of the Nb_5_Si_3_ phase remains unchanged, but its content and continuity increase. Meanwhile, compared with the Nb_3_Si phase, its decomposition product Nb_5_Si_3_ phase shows better fracture toughness [43]. Thus, the fracture toughness of the HP Nb-16Si-24Ti-2Al-2Cr alloy increases continuously with the increase in sintering temperature. However, when the sintering temperature further increases to 1450 °C, the increase in the size of the Nb_5_Si_3_ phase reduces the number of phase interfaces and the chance of crack deflection, thereby resulting in a drop in the fracture toughness. In addition, there are a large number of microcracks inside the silicides near the main crack, which can release the stress concentration during bending deformation and also contribute to the high fracture toughness.

## 4. Conclusions

In this work, the effects of sintering temperature on the microstructure, mechanical properties and fracture behavior for the HP Nb-16Si-24Ti-2Al-2Cr alloy were investigated. The major conclusions are drawn as follows:

(1) The alloys sintered at temperatures below 1400 °C consist of Nbss, Nb_3_Si and Nb_5_Si_3_ phases. When the sintering temperature reaches 1450 °C, the Nb_3_Si phase is completely decomposed into Nbss and Nb_5_Si_3_ phases. Meanwhile, the microstructure coarsens significantly.
(2) With the increase in sintering temperature, the fracture toughness first increases and then decreases, while the yield strength decreases slightly. The fracture toughness of the alloy sintered at 1400 °C reaches 20.2 MPa·m^1/2^, exceeding the application threshold. Meanwhile, the fracture toughness of the HP alloy is nearly 1.34 times that of the cast alloy.
(3) The main reason for the highest fracture toughness is attributed to the decomposition of the large-sized brittle Nb_3_Si phase and the formation of a fine microstructure, which greatly increases the number of phase interfaces and improves the chance of crack deflection. In addition, the reduction in the size and content of silicides also reduces their plastic constraints on the ductile Nbss phase.

## Data Availability

The raw data supporting the conclusions of this article will be made available by the authors on request.
